# Supplementary material for: Unexpected response of nitrogen deposition to nitrogen oxide controls and implications for land carbon sink
Source: Nat Commun. 2022 Jun 6;13:3126. doi: 10.1038/s41467-022-30854-y (PMC9170707; doi:10.1038/s41467-022-30854-y)
Supplement: Supplementary file 1 — Supplementary Information [file 41467_2022_30854_MOESM1_ESM.pdf]

Supplementary Information for

Unexpected responses of nitrogen deposition to nitrogen oxide controls and implications for land carbon sink

Mingxu Liu, Fang Shang, Xingjie Lu, Xin Huang, Bing Liu, Yu Song, Qiang Zhang, Xuejun Liu, Junji Cao, Tingting Xu, Tiantian Wang, Zhenying Xu, Wen Xu, Wenling Liao, Ling Kang, Xuhui Cai, Hongsheng Zhang, Yongjiu Dai, and Tong Zhu

Correspondence to: Yu Song and Tong Zhu

Email: songyu@pku.edu.cn (Y.S.) and tzhu@pku.edu.cn (T.Z.)

This PDF file includes:

Supplementary Text 1
Figures S1 to S12
Table S1 to S4
Supplementary References

Supplementary Text 1

Model evaluation

The Weather Research and Forcaset model coupled to Chemistry (WRF-Chem) was used to simulate the atmosphercic cycle of reactive nitrogen compounds, including their emissions, atmospheric transport, and dry/wet deposition to the Earth's surface. To evaluate the model fidelity, especially in the simulations of oxidized nitrogen (NO_y-N) deposition, we employed the nationwide observation networks for the nitrogen deposition fluxes (Supplementary Fig. 1) and airborne particulate nitrate concentrations (Supplementary Fig. 2).

For nitrogen deposition in China's terrestrial land, our model results were broadly in line with previous studies[1,2,3] with regard to the spatial distributions of deposition fluxes and the relative contributions of deposition forms. For example, in our simulation the annual sum of NO_y-N deposition over the Chinese terrestrial land was estimated to be 5.2 Tg N yr^{-1} in 2015, accounting for 47% of the total N deposition, with the remaining part from reduced nitrogen (NH_x-N; 5.8 Tg N yr^{-1}); and the dry and wet forms have almost equally contributions. As a comparision, Xu et al.[2] presented nation-scale in-situ measurements across 43 monitoring sites in China for the years 2010–2014; and they showed that NO_y-N contributes to 42% of the deposition averaged over all sites and the dry deposition shares 52% of the averaged deposition fluxes. The multiple-model ensemble results in MICS-Asia III[1] also showed that NO_y-N contributes to 48% of the total deposition over China and the dry forms contribute to 49%.

We compared the observed nitrogen deposition fluxes (kg N ha^{-1} yr^{-1}) in a nationwide monitoring network with the corresponding simulations. It is revealed that high NO_y-N fluxes of more than 10 kg N ha^{-1} yr^{-1} are concentrated in the Eastern China, the Southern China, and the Southwestern China, with the hotspots reaching 20 kg N ha^{-1} yr^{-1} (marked in Supplementary Fig. 1a). There were 54% of the model results having the discrepancies with the observations within a factor of two (FAC2). Similarly, the Model Inter-Comparison projet, MICS-Asia III[1], has shown the model-ensemble NO_y-N fluxes between 10–20 kg N ha^{-1} yr^{-1} in eastern China. The FAC2 of NO_y-N values for different participating models ranged from 36.1% to 60.2%. Though that the model-observation

comparison may be subject to some uncertainties in this study, in part because the model with a coarse horizontal grid resolution (50 km × 50 km) was not able to perfectly capture the influences of local souces and complex terrain on in-situ observations, our model achieved reasonable and state-of-the-art performance in characterizing the nitrogen deposition over China.

For the simulations of particulate nitrate formation, our recent studies have demonstrated that the model used in this study can well represent the chemical formation of nitrate acids and their thermodynamic partitioning between the gas and aerosol phases[4,5]. Similar to previous reports, we provided the detailed comparion of modeled nitrate concentrations with the airborne measurements at in-situ stations across China (Supplementary Fig. 2). The high nitrate concentrations in Eastern China in both our simulations and observations reflect the intensive anthropogenic nitroge oxide (NO_x) emissions and the efficient transformation of NO_x to nitrate acids via both daytime and nighttime oxidation regimes. The observed seasonal contrast of nitrate pollutions between summer and winter periods was captured by our model (Supplementary Fig. 2b and c), which represeted the enhanced formation of nitrate aerosols in winter due to favorable environmental condistions (e.g., low temperature and high humidity).

The daily observations of nitrate concentrations were compared to the model results (Supplementary Fig. 2d and e). We also calculated several statiscal indexs to indicate the model performance of nitrate pollutions. The normalized mean biases was 27% for the summer period and –29% for the winter period. More than 60% of the comparisons show the differences within a factor of two. Our resuls were actually much bettter than other recent nitrate simulations in China[6]. To sum up, we gave a thorough model evaluation using those in-situ measurements of atmospheric reactive nitrogen compounds and their depostion fluxes to the land. The evaluation results provide a high confidence in the further analysis of the response of nitrogen deposition to NO_x emission controls.

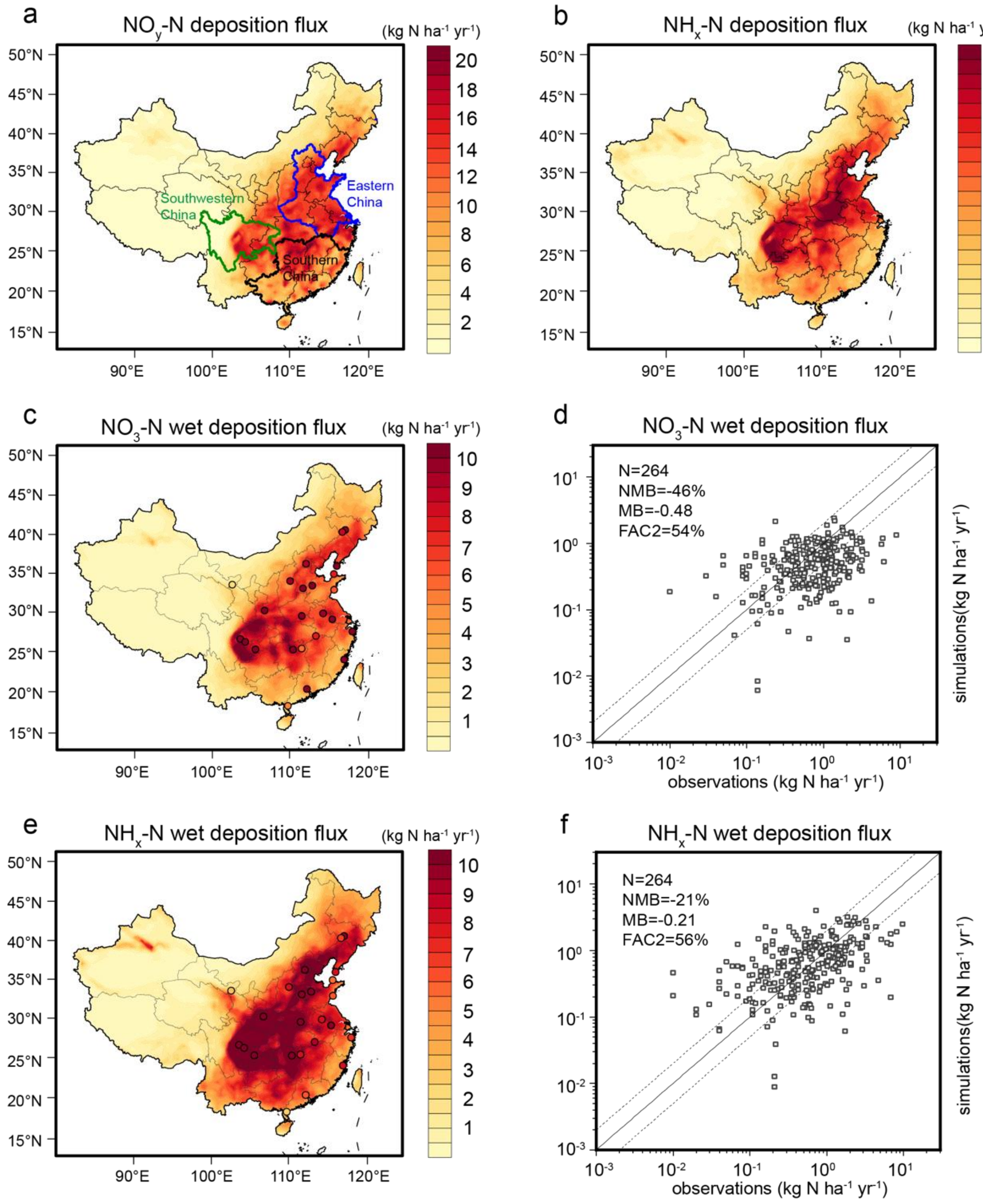

Supplementary Fig. 1 Evaluation of simulated nitrogen deposition flux against the observations of 2015 in China mainland. a Simulated spatial distribution of annual accumulated NO_y-N deposition flux in China in the Baseline case. Eastern China (marked in blue line) includes Beijing, Tianjin, Hebei, Shandong, Henan, Shanghai, Jiangsu and Anhui provinces. Southern China (marked in black thick line) includes Hunan, Jiangxi, Fujian, Guangdong and Guangxi provinces. Southwestern China (marked in green line) includes Chongqing and Sichuan provinces. **b** Spatial distribution of NH_x-N deposition flux**. c, d** Comparison of NO_3-N wet deposition fluxes with the observations at 24 sites. **e, f** Comparison of NH_x-N wet deposition with the observations. Filled circles are annually accumulated observations. The observations were derived from Xu et al.[7] databases. Three statistics indexes, normalized mean bias (NMB), mean bias (MB) and percentage of model results within a factor of two with observations (FAC2) are shown for reference. The solid 1:1 line and two dashed 1:2 and 2:1 lines are shown in the panel e and f. The map of China was reproduced from the National Geographic Information Resource Directory Service System (https://github.com/huangynj/NCL-Chinamap.git and https://www.webmap.cn/commres.do?method=result100W).

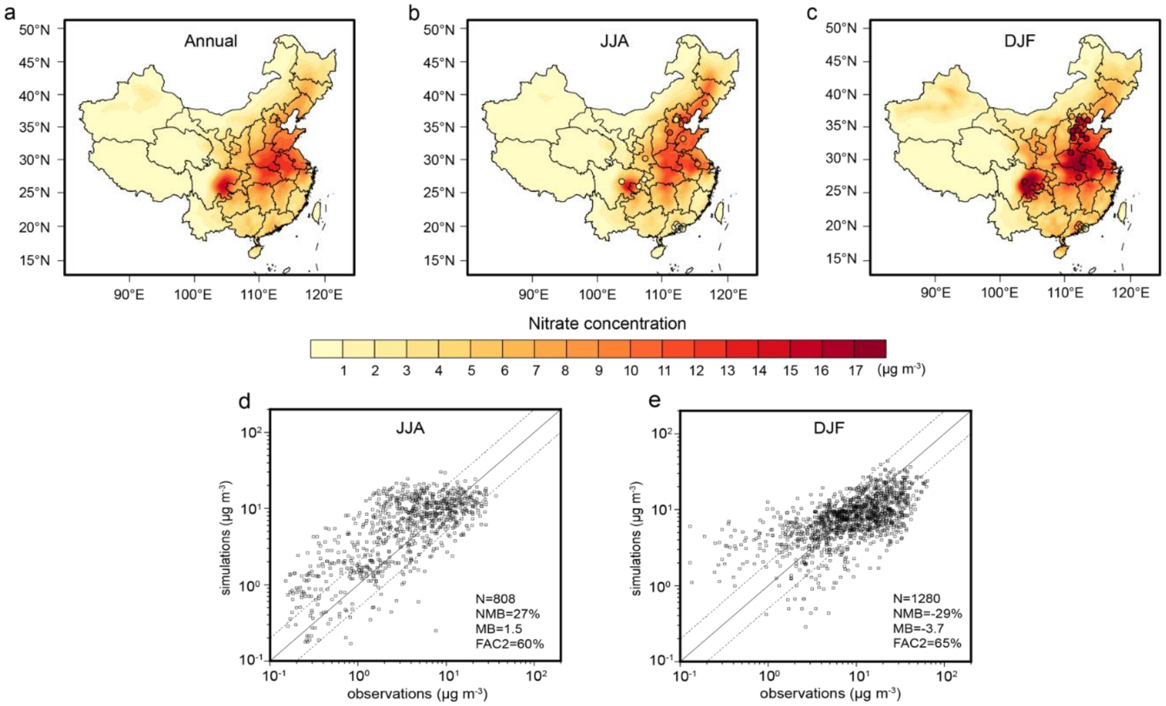

Supplementary Fig. 2 Evaluation of simulated aerosol nitrate concentration against the observations during JJA and DJF of 2015. a Spatial distribution of simulated annual mean nitrate concentration in China. **b** Spatial distribution of simulated mean nitrate concentration during JJA (June, July, and August). Filled circles are seasonally mean observations. **c** Spatial distribution of nitrate concentrations for DJF (December, January, and February). **d** Evaluation of simulated nitrate against the daily observations at 28 sites during JJA. The locations of sites are shown by the filled circles in the panel **b**. **e** Evaluation of simulated nitrate against the daily observation at 35 sites during DJF. The site locations are shown by the filled circles in the panel **c**. Description of the sites information can be seen in Liu et al.[5]. Three statistics index, normalized mean bias (NMB), mean bias (MB) and percentage of model results within a factor of two with observations (FAC2) are shown here for reference. The solid 1:1 line and two dashed 1:2 and 2:1 lines are shown. The map of China was reproduced from the National Geographic Information Resource Directory Service System (https://github.com/huangynj/NCL-Chinamap.git and https://www.webmap.cn/commres.do?method=result100W).

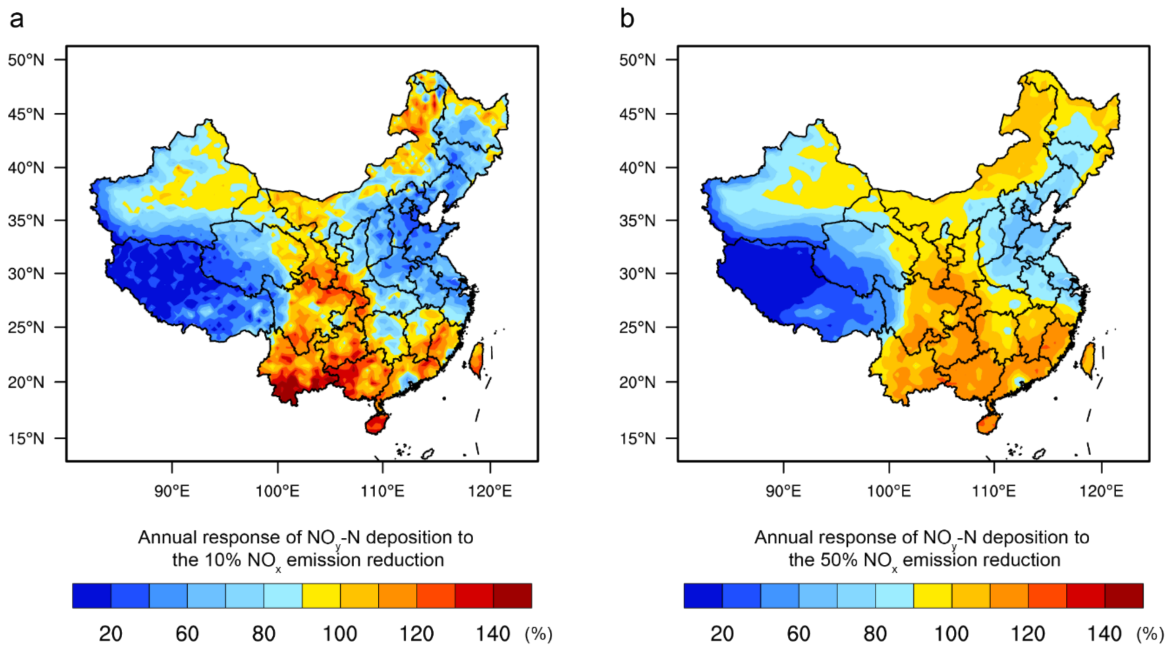

Supplementary Fig. 3 Response of NO_y-N deposition to 10% and 50% reductions of NO_x emissions. a Spatial distribution of the response of NO_y-N deposition to the 10% NO_x emission reduction. **b** The response of NO_y-N deposition to the 50% NO_x emission reduction. In this study, the response is defined as the ratio of changes in N deposition (total, NO_y-N dry, or NO_y-N wet) to changes in NO_x emissions. The map of China was reproduced from the National Geographic Information Resource Directory Service System (https://github.com/huangynj/NCL-Chinamap.git and https://www.webmap.cn/commres.do?method=result100W).

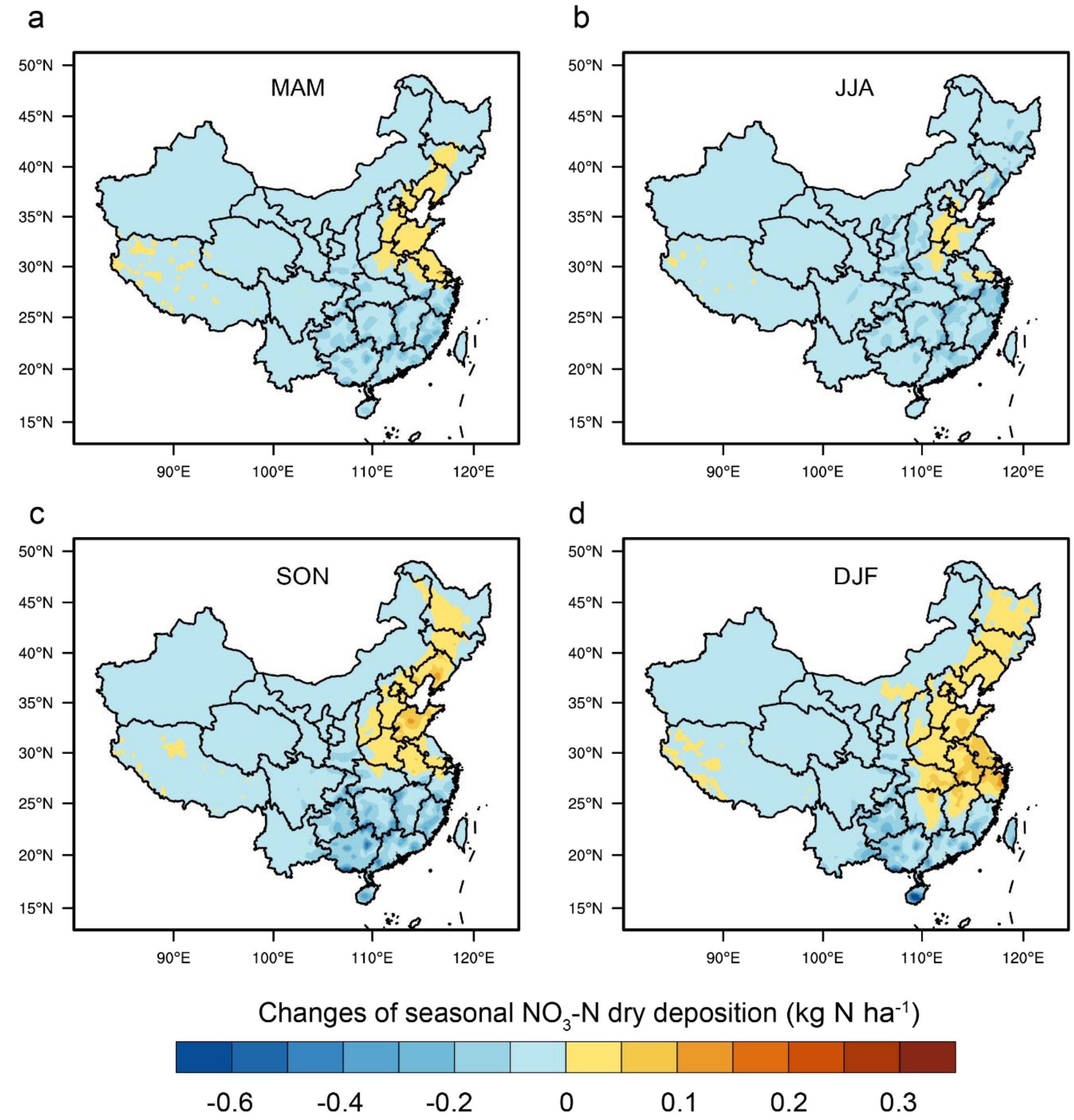

Supplementary Fig. 4 Map of changes (kg N ha^{-1} yr^{-1}) in seasonal NO_3-N dry deposition in response to a nationwide 10% reduction of NO_x emissions relative to the Baseline case. Four different seasonal periods are shown, i.e., **a** MAM (March, April, and May), **b** JJA (June, July, and August), **c** SON (September, October, and November), and **d** DJF (December, January, and February). Positive values denote enhancement of deposition fluxes. The map of China was reproduced from the National Geographic Information Resource Directory Service System (https://github.com/huangynj/NCL-Chinamap.git and https://www.webmap.cn/commres.do?method=result100W).

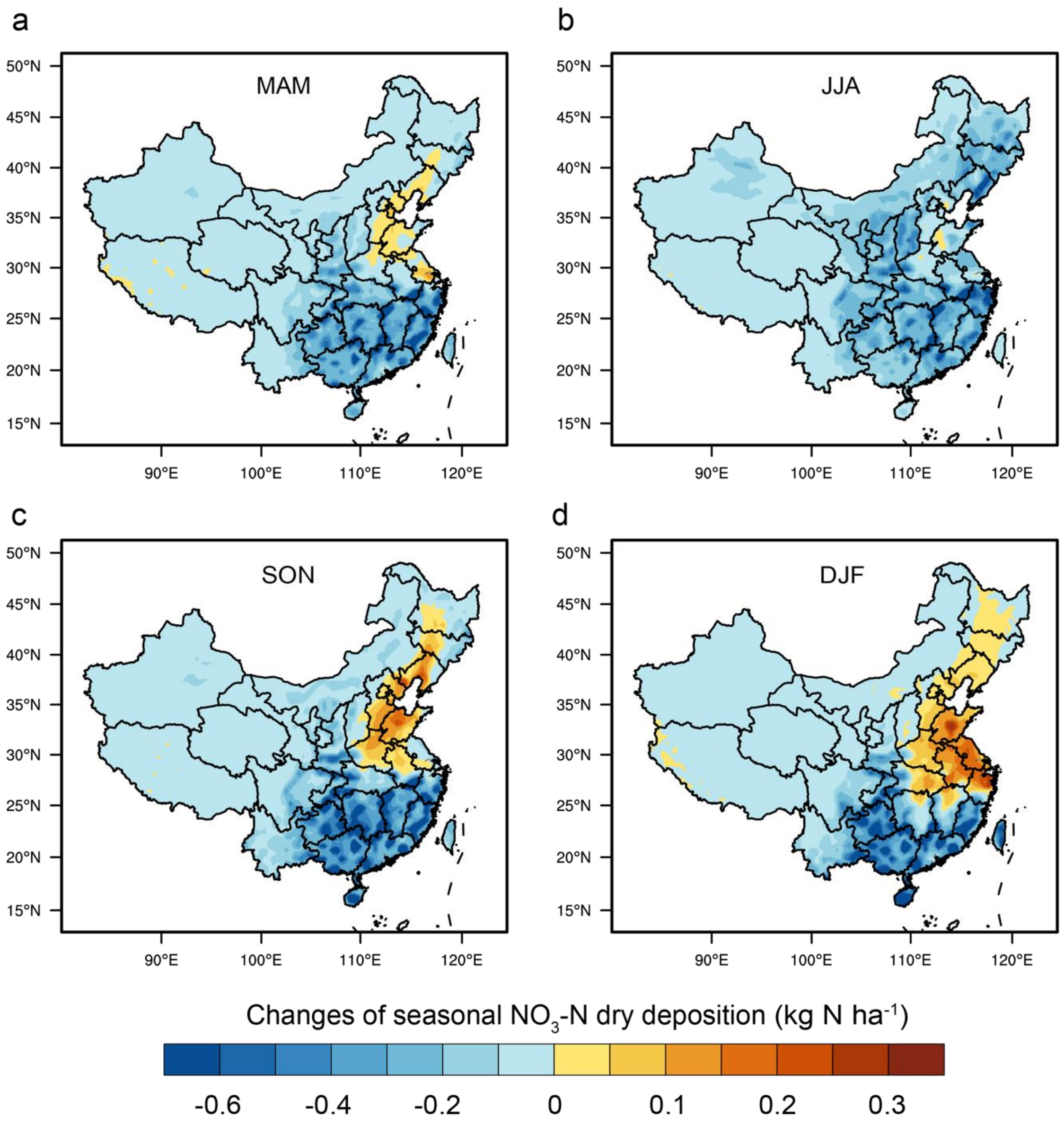

Supplementary Fig. 5 Map of the changes (kg N ha^{-1} yr^{-1}) in seasonal NO_3-N dry deposition in response to a nationwide 30% reduction of NO_x emissions relative to the Baseline case. Four different seasonal periods are shown, i.e., a MAM (March, April, and May), b JJA (June, July, and August), c SON (September, October, and November), and d DJF (December, January, and February). Positive values denote enhancement of deposition fluxes. The map of China was reproduced from the National Geographic Information Resource Directory Service System (https://github.com/huangynj/NCL-Chinamap.git and https://www.webmap.cn/commres.do?method=result100W).

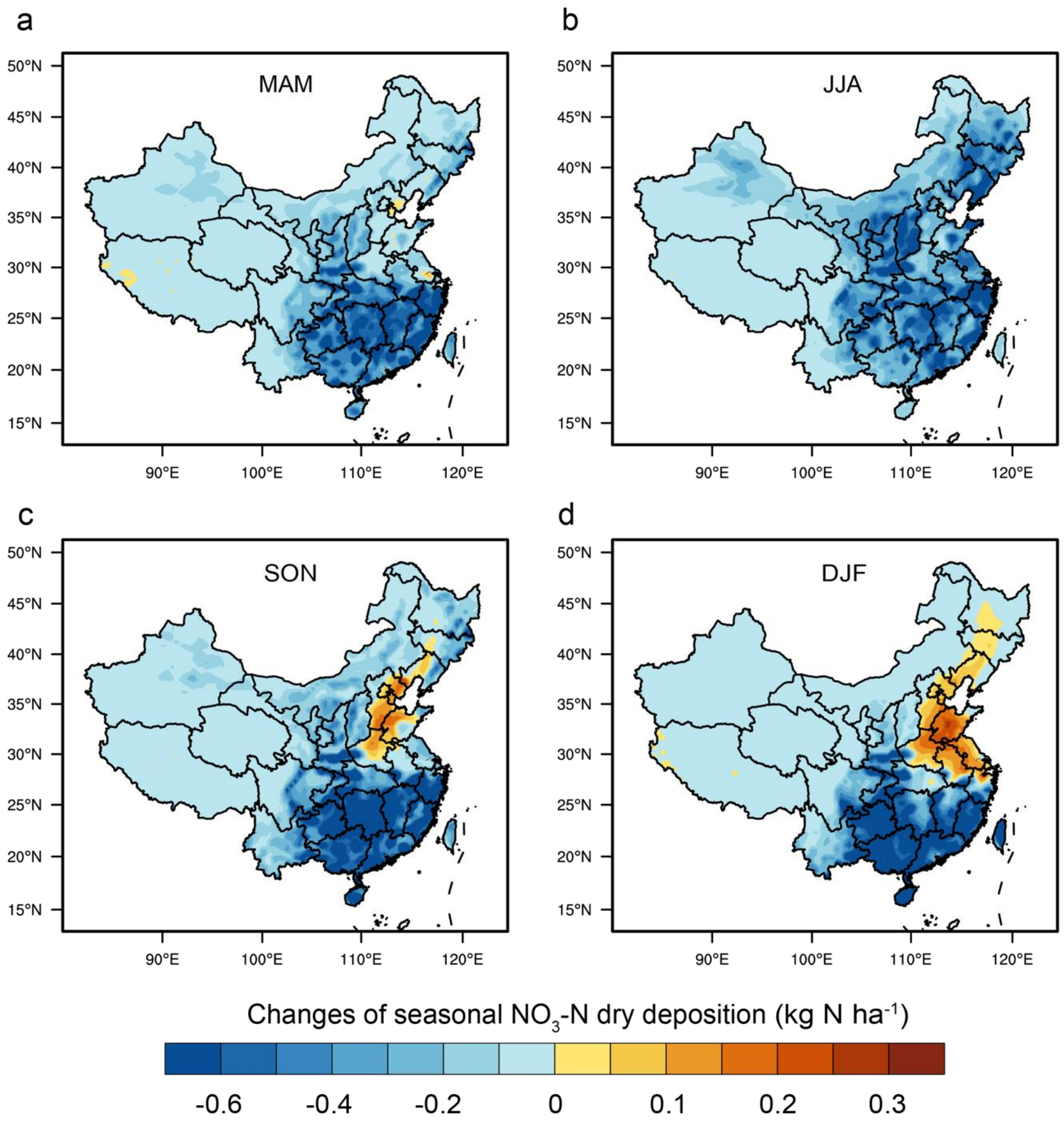

Supplementary Fig. 6 Map of changes (kg N ha^{-1} yr^{-1}) in seasonal NO_3-N dry deposition in response to a nationwide 50% reduction of NO_x emissions relative to the Baseline case. Four different seasonal periods are shown, i.e., **a** MAM (March, April, and May), **b** JJA (June, July, and August), **c** SON (September, October, and November), and **d** DJF (December, January, and February). Positive values denote enhancement of deposition fluxes. The map of China was reproduced from the National Geographic Information Resource Directory Service System (https://github.com/huangynj/NCL-Chinamap.git and https://www.webmap.cn/commres.do?method=result100W).

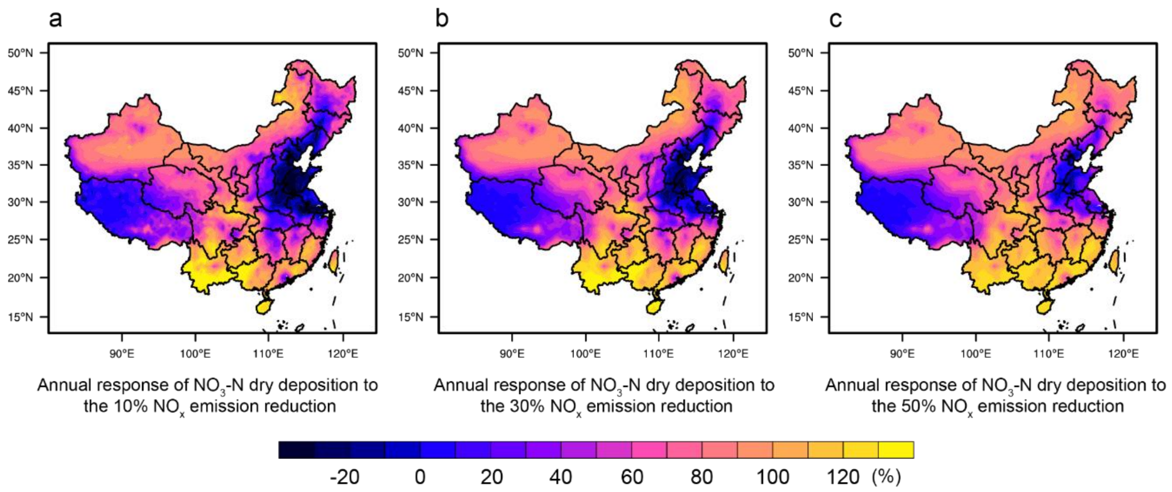

Supplementary Fig. 7 Response of NO_3-N dry deposition to 10%, 30% and 50% reductions of NO_x emissions in China's land. a Spatial distribution of the response of NO_3-N dry deposition to the 10% NO_x emission reduction. **b** The response of NO_3-N dry deposition to the 30% NO_x emission reduction. **c** The response of NO_3-N dry deposition to the 50% NO_x emission reduction. The map of China was reproduced from the National Geographic Information Resource Directory Service System (https://github.com/huangynj/NCL-Chinamap.git and https://www.webmap.cn/commres.do?method=result100W).

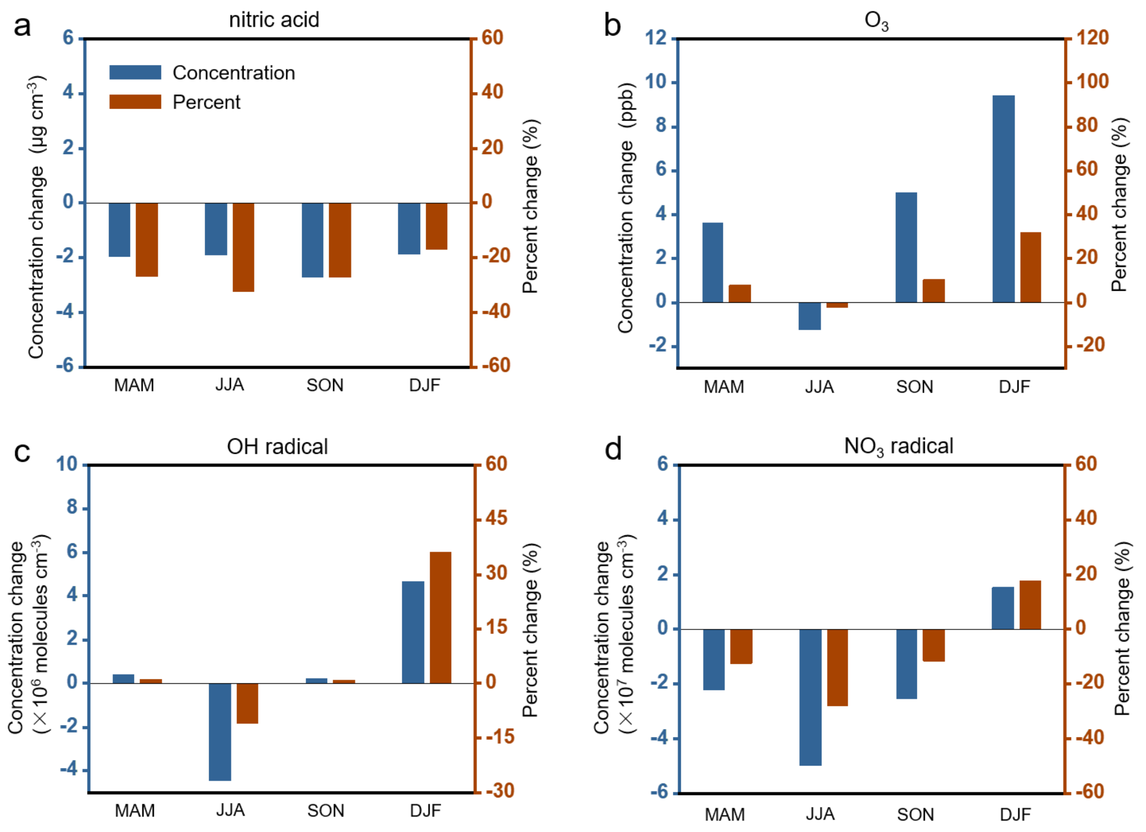

Supplementary Fig. 8 Seasonal averaged changes in near-surface nitric acid, O_3, NO_3 radical and OH radical concentrations in Southern China under a 30% NO_x emission reduction. Both absolute and relative changes were calculated for **a**. nitric acid, **b**. O_3, **c**. NO_3 radical, and **d**. OH radical. Four different seasonal periods are shown, i.e., MAM (March, April, and May), JJA (June, July, and August), SON (September, October, and November), and DJF (December, January, and February).

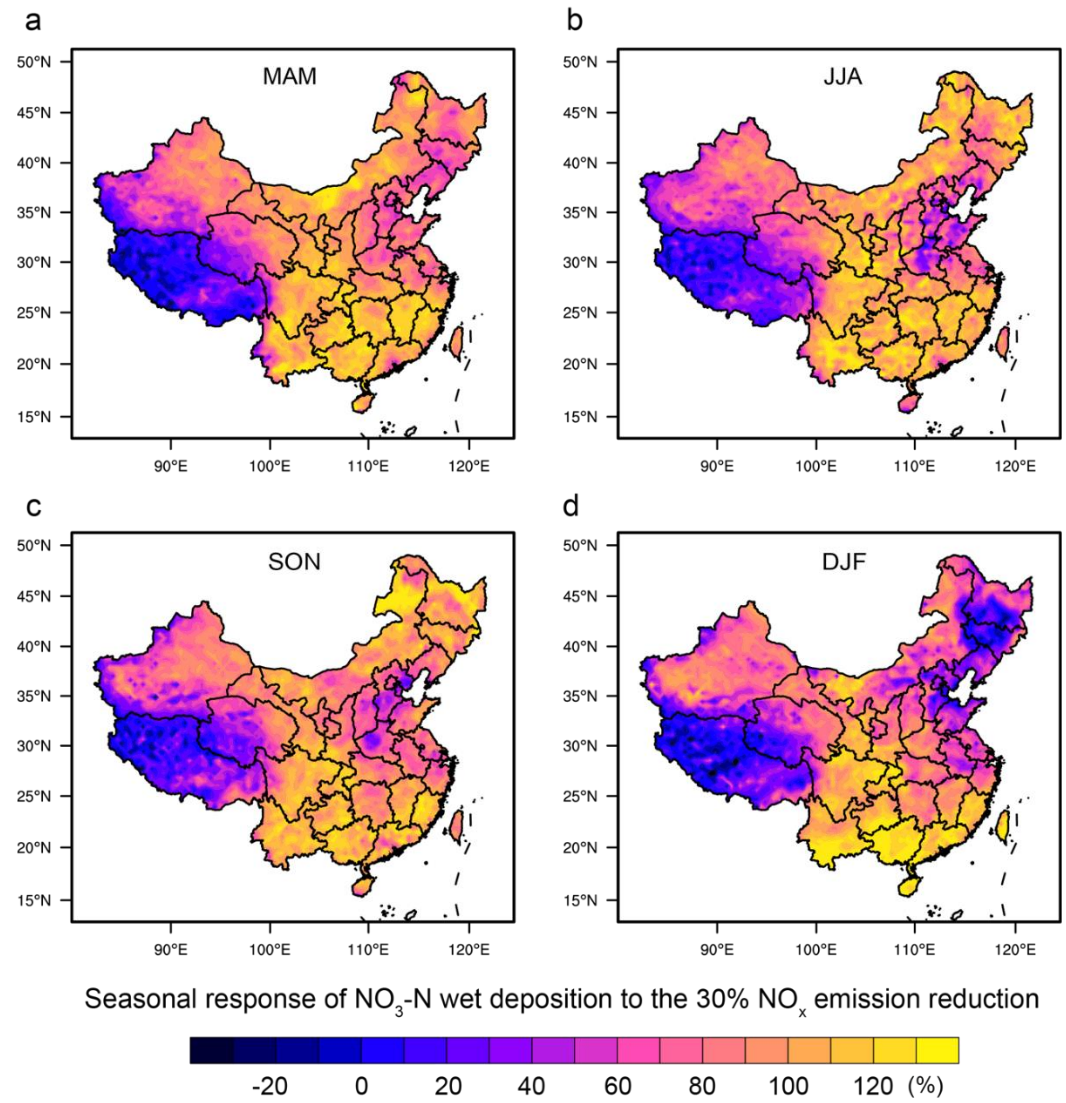

Supplementary Fig. 9 Seasonal responses of NO_3-N wet deposition to a 30% reduction of NO_x emissions. Four different seasonal periods are shown, i.e., **a** MAM (March, April, and May), **b** JJA (June, July, and August), **c** SON (September, October, and November), and **d** DJF (December, January, and February). The map of China was reproduced from the National Geographic Information Resource Directory Service System (https://github.com/huangynj/NCL-Chinamap.git and https://www.webmap.cn/commres.do?method=result100W).

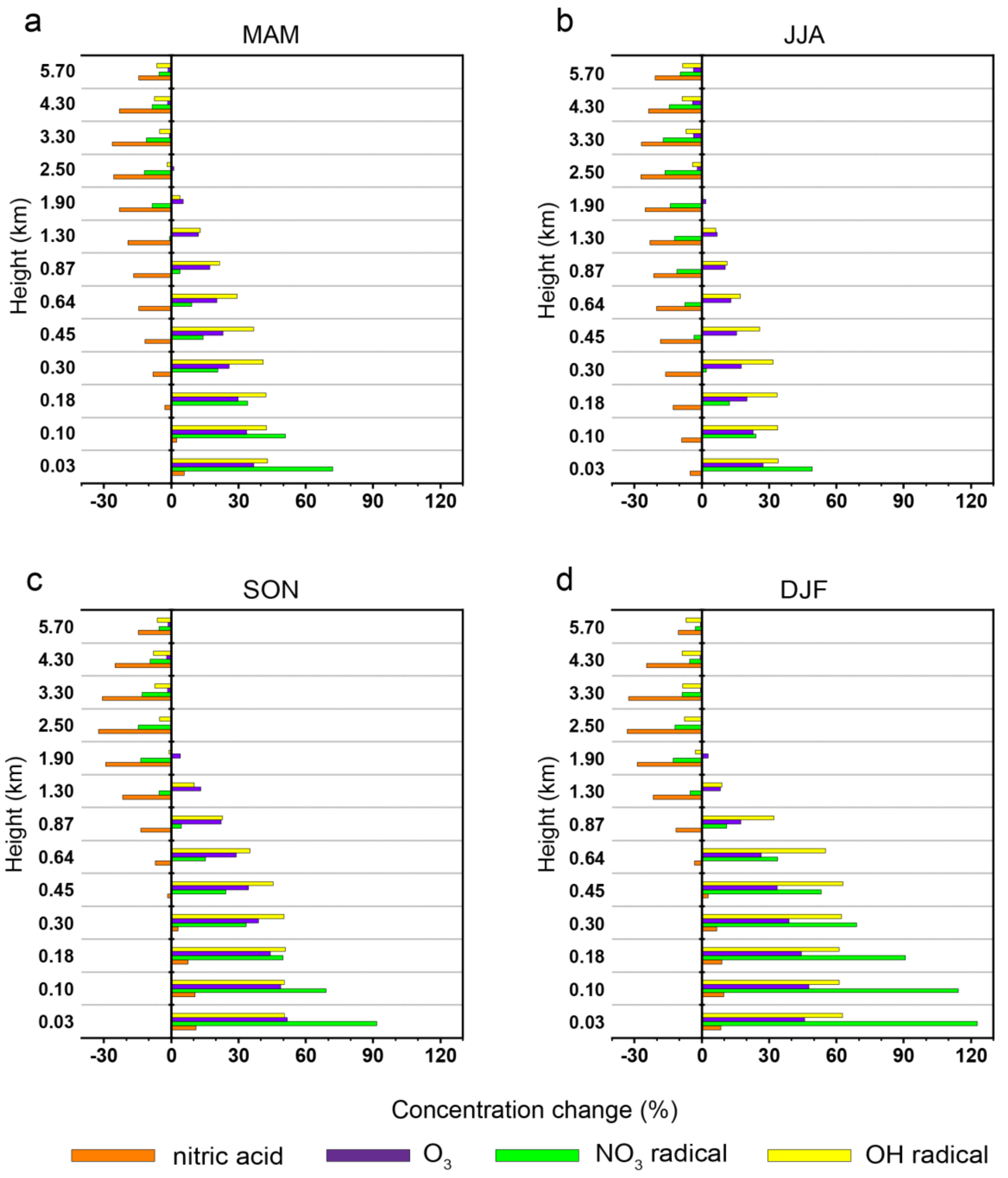

Supplementary Fig. 10 Seasonal averaged changes (%) in vertical gradient nitric acid, O_3, NO_3 radical and OH radical under a 30% NO_x emission reduction over Eastern China. Four different seasonal periods are shown, i.e., **a** MAM (March, April, and May), **b** JJA (June, July, and August), **c** SON (September, October, and November), and **d** DJF (December, January, and February).

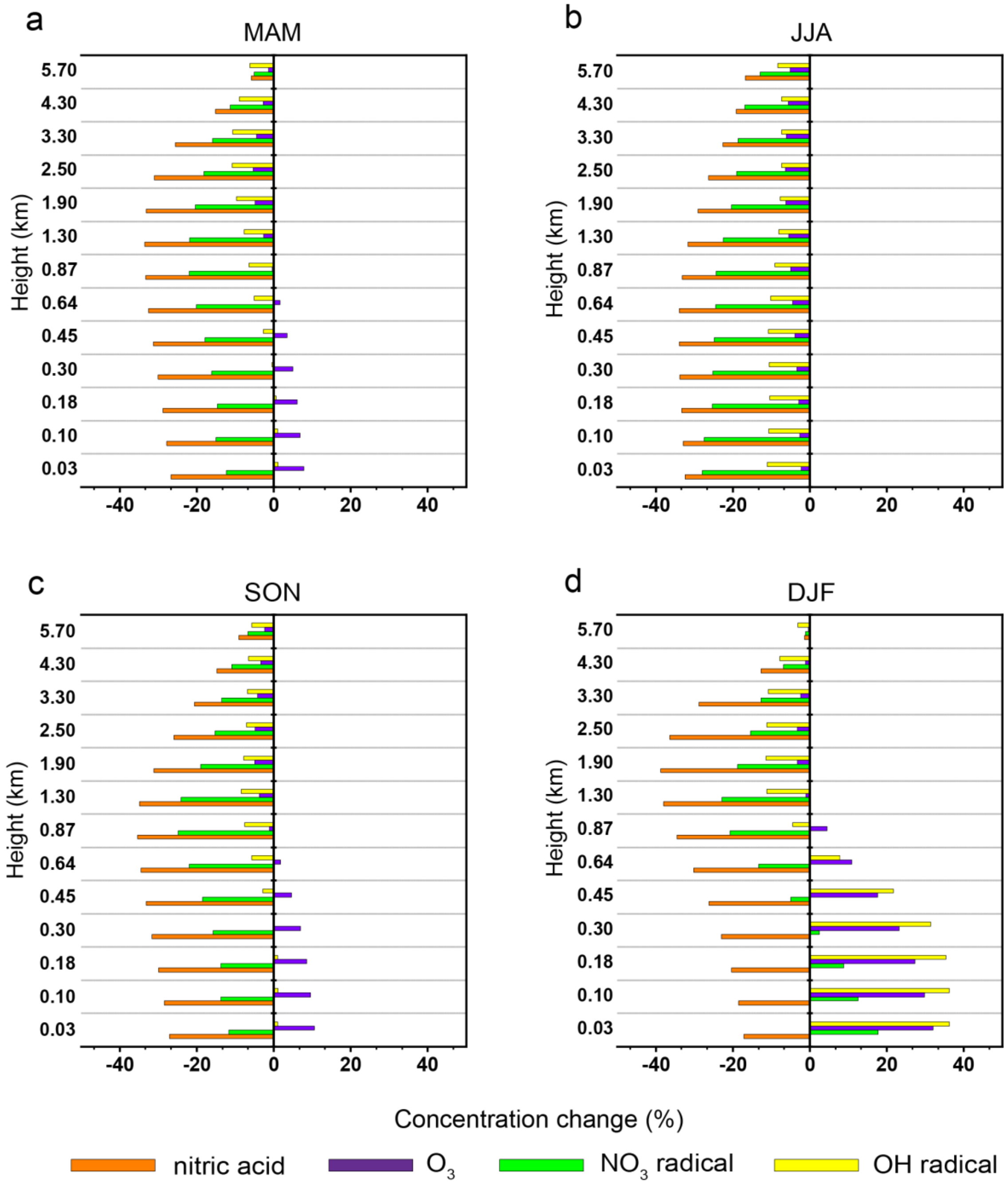

Supplementary Fig. 11 Seasonal averaged changes (%) in vertical gradient nitric acid, O_3, NO_3 radical and OH radical under a 30% NO_x emission reduction over Southern China. Four different seasonal periods are shown, i.e., **a** MAM (March, April, and May), **b** JJA (June, July, and August), **c** SON (September, October, and November), and **d** DJF (December, January, and February).

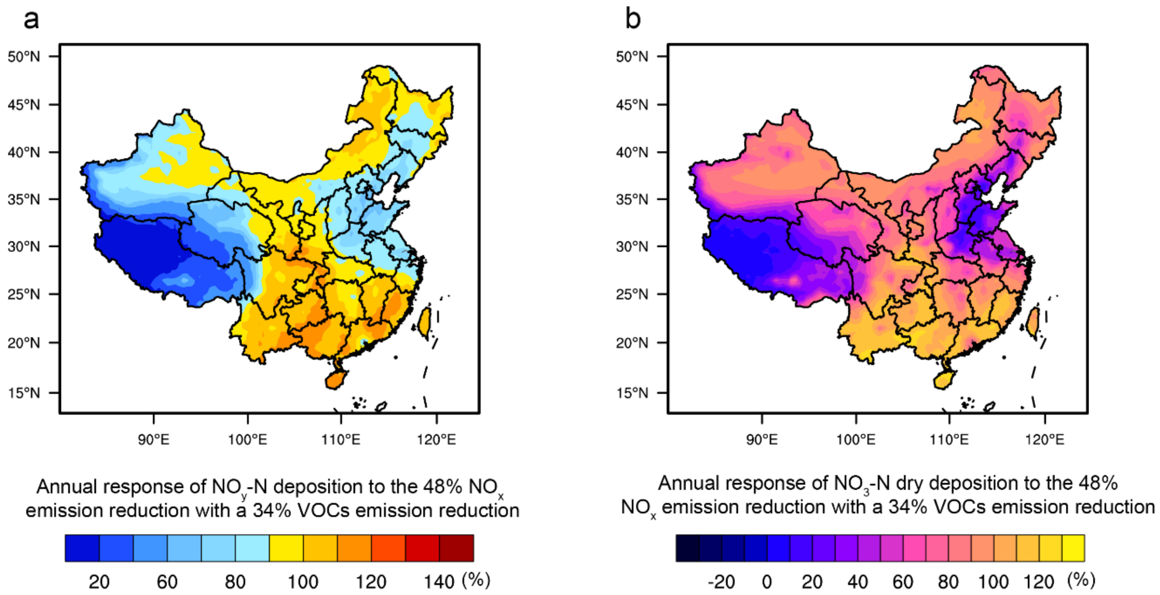

Supplementary Fig. 12 Response of NO_y-N and NO_3-N dry deposition to reduction of NO_x emissions with a 34% reduction of VOCs in China. a Response of NO_y-N deposition to the 48% NO_x emission reduction with a 34% reduction of VOCs. **b** Response of NO_3-N dry deposition in the same case. The map of China was reproduced from the National Geographic Information Resource Directory Service System (https://github.com/huangynj/NCL-Chinamap.git and https://www.webmap.cn/commres.do?method=result100W).

Supplementary Table 1 Simulation experiments by WRF-Chem

Case	Description
Baseline	One simulation was performed using the anthropogenic emissions of all species and meteorological input data for the year 2015
RED10	The same with the Base case but reducing anthropogenic NOx emissions by 10%
RED30	The same with the Base case but reducing anthropogenic NOx emissions by 30%
RED50	The same with the Base case but reducing anthropogenic NOx emissions by 50%
RED50_VOCs	The same with the Base case but reducing anthropogenic NOx emissions by 48% and VOCs emissions by 34%
Base_meteo	Five-year simulation with fixed emissions at 2015 and the meteorological conditions for the years 2011-2015
RED30_2011	The same with the RED30 but using the meteorological conditions for 2011
N_2O_5_off	The formation pathways of NO_3 radical and N_2O_5 were turned off in the simulation
EC_off	The anthropogenic emissions in the Eastern China were turned off.
SC_off	The anthropogenic emissions in the Southern China were turned off.
Other_off	The anthropogenic emissions in the other region were turned off.

Supplementary Table 2 Comparison of simulated surface concentrations of O_x (O_x=O_3+NO_2) with the observations at air quality stations during winter of 2015 across China. The observation data were collected from the China Environmental Monitoring Center (available at: https://www.aqistudy.cn/historydata/).

City Name	Latitude	Longitude	Observation ($\mu g\ m^{-3}$)	Simulation ($\mu g\ m^{-3}$)
Beijing	40.3	116.8	106.0	90.5
Shijiazhuang	38.04	114.5	102.0	82.1
Shenyang	41.8	123.43	98.3	90.7
Changchun	43.89	125.32	109.3	91.5
Haerbin	45.76	126.64	112.0	95.8
Taiyuan	37.86	112.55	68.7	78.9
Jinan	36.67	117	109.7	90.6
Zhengzhou	34.3	113.65	110.3	86.6
Nanyang	32.99	112.54	89.7	76.3
Wuhan	31	114.31	111.0	86.7
Shanghai	31.23	121.47	121.7	97.5
Nanjing	32.07	118.78	111.3	93.4
Hangzhou	30.29	120.15	109.0	105.6
Xi'an	34.27	108.93	84.7	90.9
Chengdu	30.67	104.06	101.3	105.8
Chongqing	29.53	106.5	76.7	106.3
Nanchang	28.68	115.89	83.3	91.3
Changsha	28.2	113	98.7	90.5
Hefei	31.86	117.28	74.0	92.8
Guangzhou	23.5	113.5	113.3	96.3

Supplementary Table 3 The simulated nitrogen deposition over China in 2015.

	Deposition process	Deposition（Tg N yr^{-1}）
**NO_y**	Total	5.20
	NO_3-N	4.33
	Gas-phase NO_3-N dry	1.49
	Particulate NO_3-N dry	0.3
	NO_3-N wet	2.54
	NO_x	0.74
	N_2O_5	0.06
	PAN	0.07
**NH_x**	Total	5.78
	NH_x-N dry	1.74
	NH_x-N wet	4.04

Supplementary Table 4 Percent changes in annual NO_y deposition by the modelled variations of meteorological conditions from 2011 to 2015. The results were derived from the WRF-Chem simulations over China with constant emissions but year-to-year meteorological data input during 2011-2015.

Station	Change (%)
BJ	-3%
QZ	15%
SZ	-6%
YQ	10%
ZZ	-2%
ZD	28%
CD	10%
LS	24%
DL	5%
YL	12%

Supplementary References

1. Ge, B. et al. Model Inter-Comparison Study for Asia (MICS-Asia) phase III: multimodel comparison of reactive nitrogen deposition over China. *Atmos. Chem. Phys.* **20**, 10587-10610 (2020).

2. Xu, W. et al. Quantifying atmospheric nitrogen deposition through a nationwide monitoring network across China. *Atmos. Chem. Phys.* **15**, 12345-12360 (2015).

3. Zhao, Y. et al. Atmospheric nitrogen deposition to China: A model analysis on nitrogen budget and critical load exceedance. *Atmos. Environ.* **153**, 32-40 (2017).

4. Liu, M. et al. Rapid SO2 emission reductions significantly increase tropospheric ammonia concentrations over the North China Plain. *Atmos. Chem. Phys.* **18**, 17933-17943 (2018).

5. Liu, M. et al. Ammonia emission control in China would mitigate haze pollution and nitrogen deposition, but worsen acid rain. *Proceedings of the National Academy of Sciences* **116**, 7760 (2019).

6. Miao, R. et al. Model bias in simulating major chemical components of PM2.5 in China. *Atmos. Chem. Phys.* **20**, 12265-12284 (2020).

7. Xu, W., Zhang, L., Liu, X. A database of atmospheric nitrogen concentration and deposition from the nationwide monitoring network in China. *Sci. Data* **6**, 51 (2019).
